# Photobiomodulation-induced Differentiation of Adipose-derived Stem Cells into Neuronal Organoid-like Structures

**DOI:** 10.1007/s12035-026-05903-y

**Published:** 2026-05-20

**Authors:** Precious Earldom Mulaudzi, Heidi Abrahamse, Anine Crous

**Affiliations:** Laser Research Centre, Faculty of Health Sciences, University of Johannesburg, Doornfontein, Johannesburg, 2028 South Africa

**Keywords:** Photobiomodulation, Cell differentiation, Stem cells, Neural organoids

## Abstract

The ability to regulate stem cell differentiation into organized neural tissue remains a major challenge in regenerative medicine, particularly in the development of physiologically relevant three-dimensional (3D) organoid models. Photobiomodulation (PBM) is a new non-invasive technology for controlling cellular metabolism and differentiation using light-mediated signalling pathways; however, its role in neural organoid development remains insufficiently understood. This study investigated the effects of PBM on the differentiation of adipose-derived mesenchymal stem cells (ADMSCs) into neuronal organoid-like structures using a 3D culture system. Immortalized ADMSCs were exposed to 525 nm and 825 nm wavelengths, individually and in combination, at fluences of 5 and 10 J/cm^2^. PBM’s effects were assessed using morphological assessment, cell viability, ATP-based metabolic activity, mitochondrial membrane potential (ΔΨm), and neural gene expression. PBM treatment affected organoid morphology, metabolic activity, and mitochondrial function in a dose- and wavelength-dependent way. Low-fluence irradiation (5 J/cm^2^), especially at 525 nm, promotes stem cell maintenance and early neural development, as evidenced by enhanced expression of progenitor and neuronal markers. Higher fluence (10 J/cm^2^) inhibited early differentiation responses, but mixed wavelength therapy promoted late-stage neuronal maturation with increased *RBFOX3* gene expression. These findings indicate PBM as a viable method for controlling stem cell destiny and improving neuronal organoid formation for neuroregenerative applications.

## Introduction

The ability to regulate stem cell differentiation into organized functional neural tissue represents a major breakthrough in regenerative medicine [1, 2]. Regenerative medicine’s shift towards three-dimensional (3D) organoid systems has proven effective in mimicking key structural and functional features of the human nervous system. Organoid formation is primarily mediated by controlled differentiation that determines cell fate, cell self-organization, and cell maturation, while successful neuronal differentiation does not only depend on lineage-specific signalling cues but requires well-maintained homeostasis, such as cellular viability, regulated proliferation, and coordinated cell-to-cell communication [3, 4]. Neuronal organoids (NO’s) particularly offer a unique opportunity for investigating neurodevelopment, brain repair mechanisms, and therapeutic responses [5].

Neuronal organoid systems contain a heterogeneous neural cell population, which includes neural progenitors, neurons, and glial cells that are arranged in specific patterns depending on the differentiation condition [6]. Neural progenitor populations are commonly identified by the expression of markers such as the intermediate filament protein Nestin, encoded by the *NES* gene. Glial cells, particularly astrocytes, characterized by the expression of glial fibrillary acidic protein (GFAP) [7, 8]. Mature neuronal cells are identified by the expression of neuron-specific markers such as neuron-specific markers nuclear protein (Neu N) and Class III beta-tubulin (TUBB3) [9]. However, the ability of organoid cells to survive, proliferate, and form structured cellular networks underscores their classification as functional tissue models [10].

Functional NO's are increasingly characterized using quantitative biological measures rather than relying on molecular marker expression. Parameters such as cell viability, proliferative capacity, and metabolic activity are considered essential indicators of organoid functionality, particularly during the early and intermediate stages of differentiation [11, 12]. Sustained cell viability supports the maintenance of tissue structure, while regulated proliferation enables the growth and self-renewal required for neural network development. These foundational characteristics establish conditions that are necessary for the emergence of advanced neuronal features, including synaptic organization and electrophysiological function [12]. In this study, the term neuronal organoid-like structures is used to describe 3D cellular aggregates that exhibit features of neural differentiation but do not fully meet all criteria of fully developed organoids.

Photobiomodulation (PBM) has been widely applied in regenerative medicine to enhance wound healing, modulate inflammation [13, 14], and promote stem cell differentiation [15, 16] through mitochondrial and signalling pathway activation for directing tissue development. PBM is known to influence cellular membrane activity through its hallmarks, enhancing adenosine triphosphate (ATP) production and the activation of transcriptional pathways associated with cell survival and proliferation [17, 18]. These photobiological effects have placed PBM as a promising tool for directing stem cell differentiation and supporting tissue development in 3D culture systems. Recent advances have emphasized the importance of tailoring PBM parameters to regulate specific stages of tissue regeneration through modulation of mitochondrial activity and cellular bioenergetics [19]. Interestingly, PBM has been shown to promote neurogenic pathways such as Wnt/β-catenin, PI3K/Akt, and MAPK/ERK signalling pathways [20, 21], enhance neuronal survival [22], and improve cellular resilience [23], making it particularly relevant for NO generation.

With much research focusing on the application of PBM to guide differentiation into organoid models, the neurogenic differentiation capability of ADSCs into neuronal organoids remains insufficiently explored. Integrating PBM within a 3D organoid culture may provide a controlled and reproducible means of enhancing stem cell survival and promoting neural lineage commitment during the formation of organoids. This study aimed to validate a protocol for generating neuronal organoid-like structures from ADMSCs in a 3D culture system, while investigating PBM-induced differentiation. In addition, the study explores how PBM can be incorporated for future translational applications, with a focus on its ability to support cell survival and modulate neurogenic differentiation within a 3D microenvironment. While previous work has demonstrated the role of PBM in early neural induction and mitochondrial modulation during neurosphere formation [24], its effects on later-stage 3D neural organoid development remain insufficiently explored.

## Materials and Methods

### 2D Cell Culture and Development

Immortalized human adipose-derived mesenchymal stem cells (ADMSCs; ASC52telo hTERT, ATCC® SCRC-4000™) were used in this study. These cells have also been previously characterized within our research group in accordance with established mesenchymal stem cell criteria using stem cell markers (CD44, CD90, and CD166) [25]. ADSCs were cultured in complete medium consisting of Dulbecco’s Modified Eagle Medium (DMEM) (Sigma-Aldrich, South Africa) supplemented with 20% fetal bovine serum (FBS Superior) (Biochrom, South Africa) and 1% antibiotic solution (0.5% Penicillin-Streptomycin and 0.5% Amphotericin B, Sigma-Aldrich, South Africa). The cells were maintained in Nunc™ 75-cm^2^ culture flasks (Thermo Scientific™, USA) and incubated at 37 °C with 5% CO_2_ and 85% humidity in a Heracell™ 150i CO2 incubator (Thermo Scientific™, USA).

### Culture and Differentiation of Neuronal Organoid-like Structures

Once the cells reached 85% confluence, they were harvested from the Nunc™ 75-cm^2^ culture flasks and reseeded into a 96-well plate with an ultra-low attachment surface at a density of 45 cells/µL in neural embryoid body formation medium. The embryoid formation media consisted of DMEM, supplemented with 20% fetal bovine serum, 1% antibiotics (0.5% Penicillin-Streptomycin and 0.5% Amphotericin B solution), 4 ng/mL basic fibroblast growth factor (bFGF), and 5 µM ROCK inhibitor. The plate was centrifuged at 3000 ×g for 5 min at room temperature (rtp) to allow cell aggregation formation and then incubated under static culture conditions for 24 h to allow the compaction formation before proceeding with PBM treatment. Following PBM treatment on the sixth day, embryoid formation media was replaced with neural induction media. The neural induction media consisted of DMEM-F12 supplemented with 1% N2 supplement, 1% Glutamine Plus, 1% MEM-NEAA, and 1 µg/mL heparin. The plate was further incubated for 24 h prior to the second PBM treatment. Following the second PBM treatment on the eleventh day, neural induction media was replaced with neural organoid differentiation medium without vitamin A after neurosphere embedding in Matrigel. Initially, the Matrigel was thawed at 4 °C overnight and placed on ice. Cell media was removed and a droplets of ∼30 µL Matrigel was added to each well to allow for neurosphere to embed in the Matrigel. The Matrigel droplets were incubated at 37 °C for 60 min to allow for Matrigel to solidify with the embedded neurosphere. Following incubation, a volume of ~ 200 µL of neural organoid (NO) differentiation medium was added. The neural differentiation media was composed of DMEM-F12 mixed with equal volume of neurobasal media supplemented with the following: 0.5% N2 supplement, 0.5% MEM-NEAA, 1% Glutamine Plus, 1% Penicillin-Streptomycin, 1% N21 supplement without vitamin A, 87.5 µL 2-mercaptoethanol in DMEM-F12 (1:100), and 62.5 µL of insulin.

### Photobiomodulation Treatment

Following seeding and media change organoid (*n* = 6) were PBM treated using a 525-nm green and 825-nm near-infrared laser device at continuous output and the combination wavelength in the order of green to near-infrared at both 5 and 10 J/cm^2^ fluences. A diode light source was used and the light spot at this distance was 3.4 cm^2^. The laser parameters are shown in Table 1.

**Table 1:** Photobiomodulation treatment parameters

	Average power	Irradiance (mW/cm^2^)	Irradiation time (s)	Fluence (J/cm^2^)
825 nm	180.58 ± 2.066	19.90	251.3	5
			502.5	10
525 nm	553.89 ± 2.066	60.98	82.0	5
			164.0	10

### Morphological Changes

Morphological changes and size of NO-like structures were analyzed and recorded 24 and 72 h post irradiation (hpi) using inverted light microscope (Wirsan, Olympus CKX41) with attached microscope-connected digital camera (Olympus, SC30) that uses the Olympus CellSens imaging software program (version 2.3). Measurements were performed using Olympus CellSens software, and the diameter of the core region was used as a standardized metric.

### Biochemical Analysis on Effects of PBM

All biochemical assays were performed in sextuplet, and analysis was conducted on days 13 and 15. Results are represented relative to the control*.*

#### Cell Viability Using Live/Dead Assay

The live/dead assay is a fluorescence-based viability assay that is used to differentiate between live and dead cells based on cellular membrane integrity. The NO-like structures were washed thrice with 1X PBS and stained simultaneously with 1 µg/mL of acridine orange (AO) and ethidium bromide (EtBr) for 5 min in PBS on an orbital shaker at rtp. Following incubation, the NO-like structures were washed thrice with 1X PBS and visualized using Alexa fluor 488 and EtBr channels under a confocal microscope (Leica SP5) and a fluorescence microscope (Leica DM IL LED) coupled to a camera Leica DFC 345 FX. Digitalized images were captured using Leica LAS V4.0 software.

#### ATP Metabolic Activity Assay

Cellular metabolic activity of 3D aggregates was evaluated using the CellTiter-Glo® 3D ATP (G9681, Promega) luminescence assay kit. Briefly, NO-like structures were removed from the incubator and brought to rtp for 30 min, then transferred in 100 µL of media into a 96-well opaque plate, which was followed by adding 100 µL of CellTiter-GLo® 3D reagent. The NO like structures were mixed on an orbital sharker for 5 min to induce lysis and incubated for 25 min at rtp in the dark to stabilize the luminescence signal. The luminescent signal was measured using the VICTOR Nivo® multimode plate reader (PerkinElmer, HH35940080 EN), and the signal was recorded in relative light units (RLUs).

#### Evaluation of Mitochondrial Membrane Potential (ΔΨm)

The MitoProbe JC-10 assay (MAK159, Sigma-Aldrich) was employed to measure the cellular mitochondrial membrane potential of NO-like structures. Following PBM treatment, NO-like structures were treated with the JC-10 dye loading solution, prepared by adding 100 µL of 100 × JC-10 to 10 mL of assay buffer. A volume of 100 µL loading solution was then added to the NO-like structures and incubated for 60 min at 37 °C protected from light in the incubator. Following incubation, a volume of 50 µL of assay buffer was added to each NO-like structure. ΔΨm was quantitatively measured by fluorescence ratio using excitation/emission wavelengths of 490/525 nm and 540/590 nm using a VICTOR Nivo® multimode reader (Perki-nElmer, HH35940080 EN). The red/green fluorescence intensity ratio was used to assess mitochondrial membrane potential (ΔΨm), with increased red fluorescence indicating mitochondrial membrane depolarization and increased green fluorescence indicating membrane polarization.

#### Gene Expression on Transdifferentiation

The expression levels of neural marker genes were assessed on day 15, representing a late stage of differentiation using quantitative real-time PCR (qRT-PCR) analysis. Earlier stages of neural induction and gene expression dynamics (day 0–11) have been previously characterized by our group in a recent study by Mulaudzi and co-workers [17, 24]. In this process, neurospheres were first lysed, and total RNA was extracted using the Zymo Mini Prep RNA isolation kit. This RNA was then converted to complementary DNA (cDNA) using the NEB Superscript cDNA synthesis kit. Ribosomal 18S served as housekeeping genes, providing a stable reference for comparison with neural markers, including CD90, *NSE* gene (encoding Nestin protein), *GFAP* gene (encoding glial fibrillary acidic protein), *TBR2* gene (encoding TBR2), *TUBB3* gene (encoding ß-Tubulin III), and *RBFOX3* gene (encoding NEUN protein). mRNA quantification was measured using SyBr Green according to the manufacture’s cycling conditions. The relative mRNA levels were calculated using the 2^(−ΔΔCt)^ method to quantify gene expression levels. The forward and reverse primer sequences for both the neural markers and housekeeping genes are detailed in Table 2.

**Table 2:** qPCR gene expression

Description	F-primer	R-primer
18 S	CGCGCGCTACACTGATGTATTCAA	TACAAAGGGCAGGGACGTAGTCAA
*NSE* gene (Nestin)	AGAGAGCGTAGAGGCAGTAA	GGTGCTTGAGTTTCTGGAGAT
CD 44	GACAGCGTGTACCTGACTTTAT	CTCGATAAAGGGTGGGCTTATT
*RFBOX3* gene (Neu N)	GCGGCTACACGTCTCCAACATC	ATCGTCCCATTCAGCTTCTCCC
*TBR2* gene (TBR2)	CCCAGACCCAACCTTTCC	TTCTGGAGGTCCATGGTAGT
TUBB3 gene (Beta III Tubulin)	CGCCCTCCTGCAGTATTTATGG	AGGCCTGGAGCTGCAATAAG
GFAP gene (Glial Fibrillary Acidic Protein)	GCCTCTGGATTGTGGGAATTA	GGCCTTTAGAAATGGGACAAAG

### Statistical Analysis

All experimental data are represented as means ± standard error of the mean. For each experimental condition, at least six biological replicates were analyzed per experiment (*n* = 6), where each replicate represents an individual organoid-like structure. These structures were generated from the same cell line and passage within each experiment. All acquired data were statistically analyzed using GraphPad Prism (version 10.1.1) software to determine statistical significance. Kolmogorov-Smirnov tests were used to evaluate whether the data is parametric or nonparametric. For comparisons involving multiple groups and variables, two-way analysis of variance (ANOVA) was performed, followed by Tukey’s multiple comparisons post hoc test to identify significant differences between groups. Statistical significance was set at *p* < 0.05 and was also defined as *p < 0.05, **p < 0.01, ***p < 0.001 and ****p < 0.0001.

## Results and Discussion

### Morphology

**Fig. 1:**
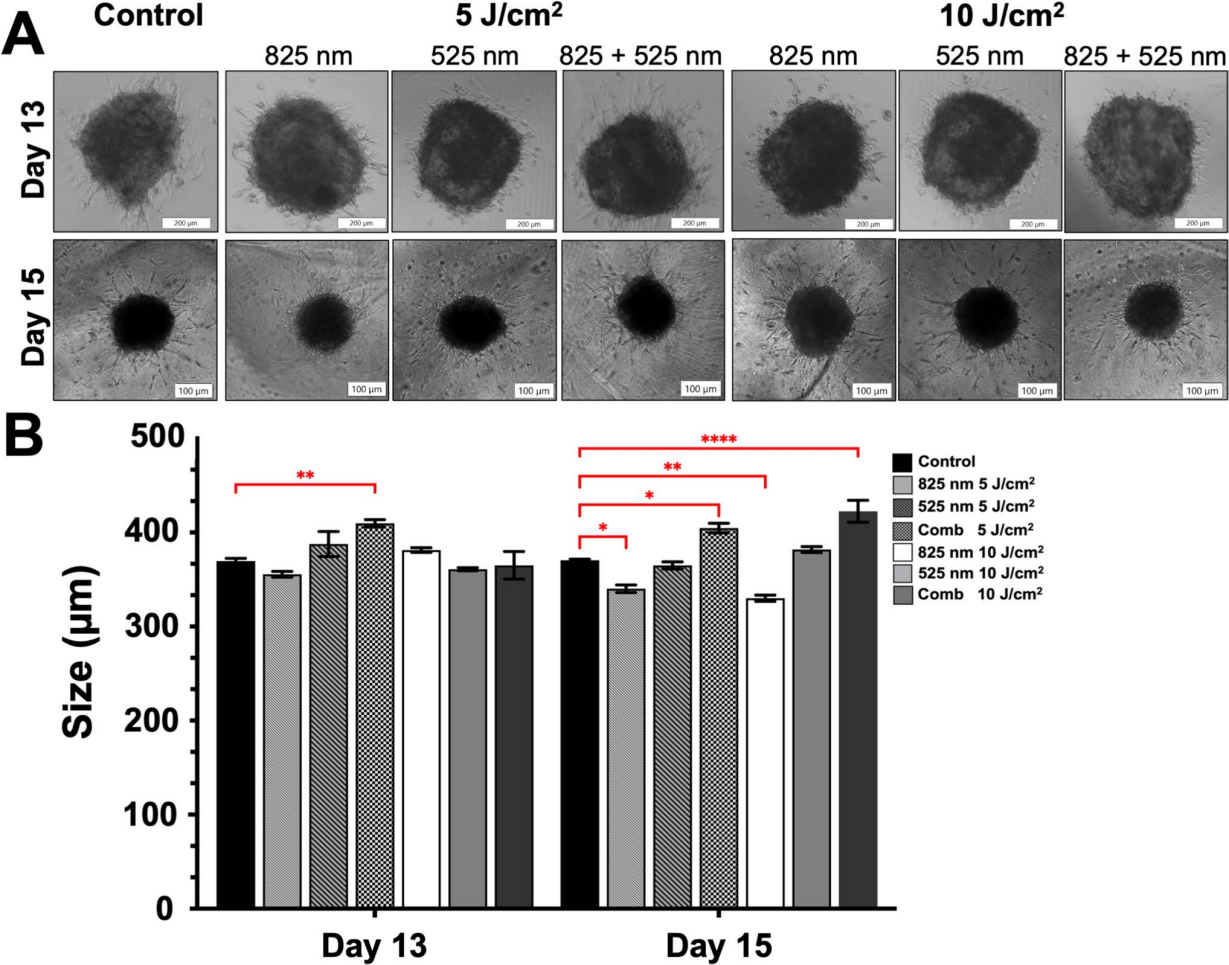
**A** Representative brightfield images of neuronal organoid-like structures embedded in Matrigel following PBM treatment, illustrating morphological changes and cellular outgrowth at day 13 (scale bar 200 µm) and day 15 (scale bar: 100 µm). Images demonstrate increased cellular expansion and neurite-like projections overtime. **B** Quantitative analysis of NO-like structure size analysis measuring the diameter of PBM-treated NO-like structures and the control. Data are presented as mean ± SEM. Statistical significance is indicated as *p < 0.5, **p < 0.01, ****p < 0.0001

Morphology was evaluated using an inverted light microscope in Matrigel. Following Matrigel embedding, organoid-like structures (Fig. 1A) developed a small population of migratory, fibroblast-like cells observed by day 13; however, by day 15, an increase in expansion of the neuron-like cell structure was observed to have increased in all groups as well as the control. The migration away from the core structure supports neuroepithelial bud outgrowth by relieving local cellular crowding or altering the microenvironment in favor of neural tissue expansion [3]. The organoid exhibited a deviation from typical neuroepithelial bud formation, instead showing extended cell processes indicative of a shift toward direct neural differentiation. This supports previous work suggesting that, rather than organizing into structured neuroepithelium, the cells prematurely committed to a differentiated neural fate [3, 26].

Size analysis on NO-like structures was conducted by measuring the diameter of the core region (Fig. 1B). Measurements conducted by day 13 showed a significant increase in size when PBM treated with the combination (825/525 nm) wavelength at 5 J/cm^2^ (*p* < 0.01) and indicates the largest organoid size. Over time, by day 15, a significant increase in diameter was observed in the combination (825/525 nm) treatment at both 5 J/cm^2^ (*p* < 0.05) and 10 J/cm^2^ (*p* < 0.0001) showing the largest increase. However, PBM at 825 nm treatment at both 5 J/cm^2^ (*p* < 0.05) and 10 J/cm^2^ (*p* < 0.01) showed a significant decrease in size when compared to the control.

### Cellular Viability

**Fig. 2:**
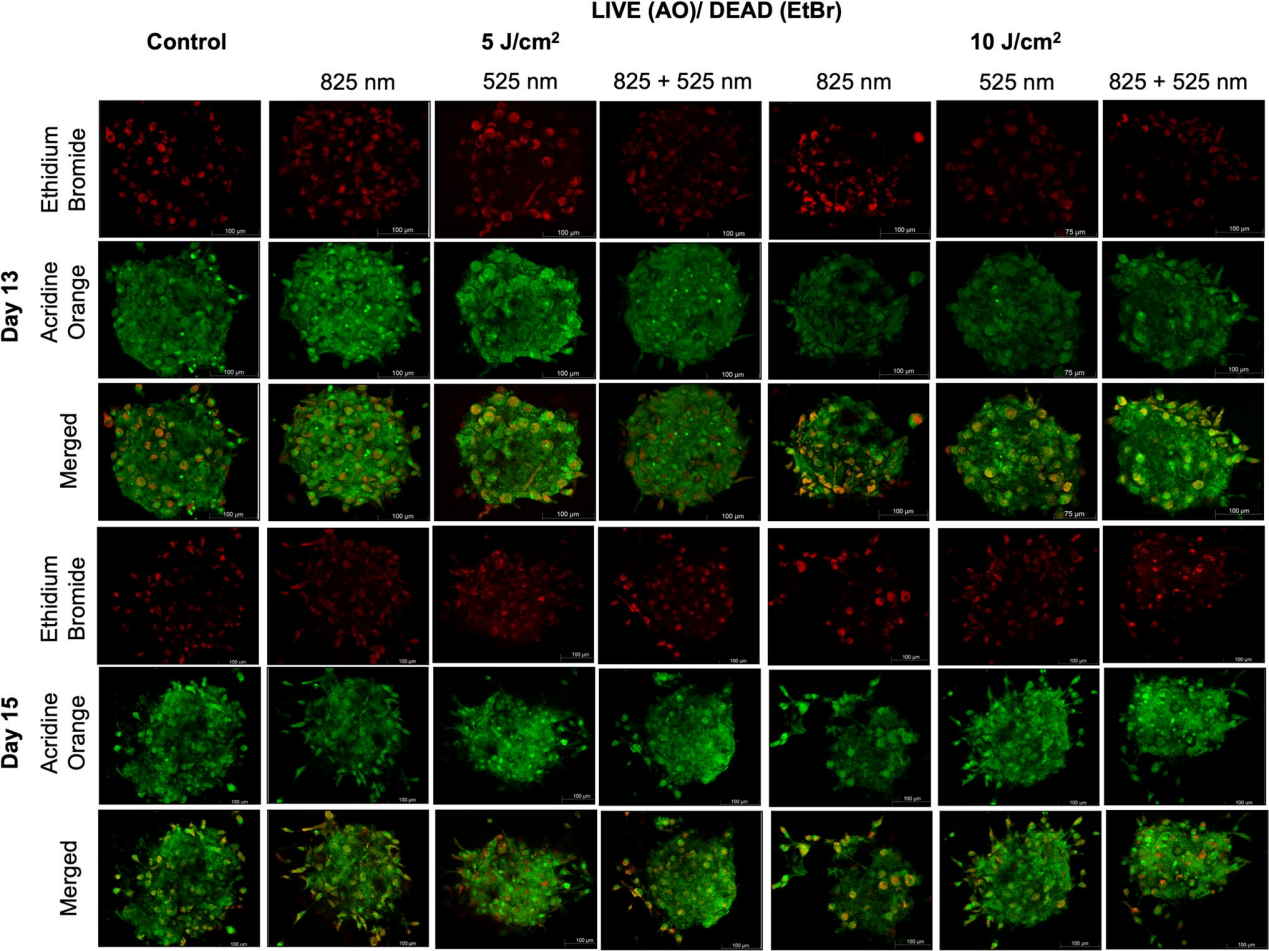
Live/dead assay of neuronal organoid-like structures using acridine orange and ethidium homodimer-1 to assess cell membrane integrity on day 13 and day 15. Representative organoid images stained for viability reveal widespread green fluorescence (AO-positive live cells) with sparse red fluorescence (EtBr-1-positive dead cells) in PBM-treated NO-like structures

Cellular viability is a key indicator of the proportion of living, metabolically active cells within a given population and serves as a critical indicator of cell health, particularly in 3D culture, where nutrient diffusion and oxygen gradients can significantly impact cell survival [27, 28]. Live/dead assay used in this study provides a qualitative assessment of cell viability based on membrane integrity, allowing for visualization of viable and non-viable cells within the 3D organoid structure. Following PBM treatment, by day 13 (Fig. 2), cellular viability remained high in all treatment groups and the control. Similarly, by day 15, cellular viability was increased and the presence of viable cells increased. The strong green fluorescence and minimal red signal indicated intact cellular membrane integrity and low levels of necrosis within the NO-like structures. While the presence of membrane-compromised organoid-like structures was observed, previous work by Mulaudzi and co-workers showed that PBM does not induce changes in cell health, but the 3D cell structure of a spheroid form increases cell membrane integrity [29]. This results also support the hallmark of PBM, that PBM is not a harmful application on cell viability, as cell viability remained increased, and this was supported by previous work by Mulaudzi et al. (2025) in a study that confirmed cell viability remains unaffected by PBM irrespective of the treatment introduced [17].

### Analysis of Metabolic Activity

**Fig. 3:**
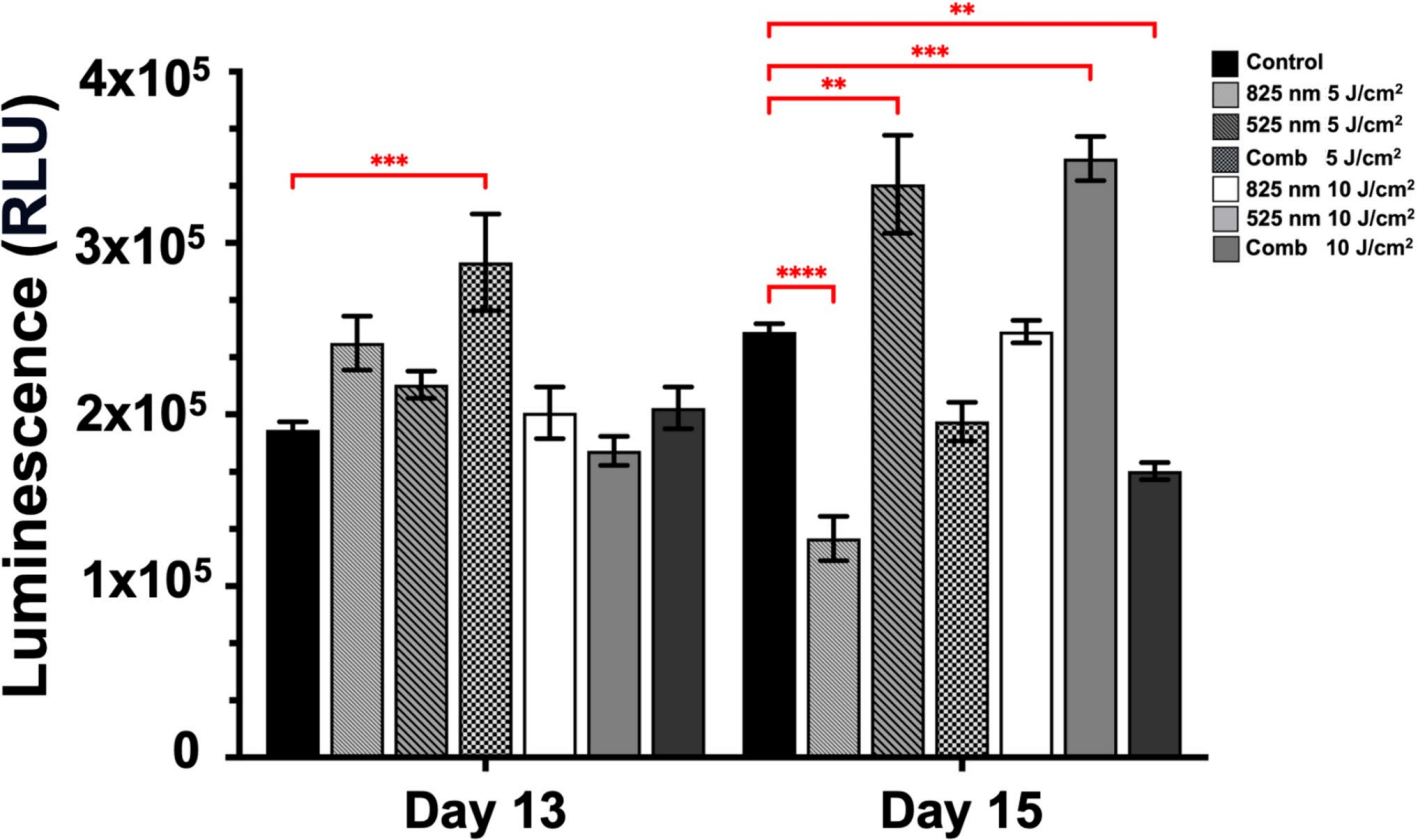
Effects of PBM on ATP production during neural differentiation. ATP levels, expressed as relative luminescence units (RLU), were measured at day 13 and day 15 following PBM treatment using 825 nm, 525 nm, or a combination of 825/525 nm wavelengths at 5 J/cm^2^ and 10 J/cm^2^, alongside untreated controls. Data are presented as mean ± SEM. Statistical significance is indicated as **p < 0.01, ***p < 0.001, ****p < 0.0001

ATP production (Fig. 3) was assessed at day 13 and day 15 to evaluate the effects of PBM on cellular metabolic activity during neural differentiation. At day 13, PBM treatment resulted in a significant increase in ATP levels compared to control conditions. The combination treatment at 5 J/cm^2^ produced the highest ATP levels, showing a significant increase relative to other treatment groups (*p* < 0.001). Both 825 nm and 525 nm treatments also elevated ATP production compared to the control, though to a lesser extent. By day 15, the 525 nm treatment resulted in the highest ATP levels and was significantly increased compared to control and other PBM conditions at 5 J/cm^2^ (*p* < 0.01) and 10 J/cm^2^ (*p* < 0.001). It was also observed that there was a significant decrease in the amount of ATP produced when NO-like structures when PBM treated with combination treatment at 10 J/cm^2^ (*p* < 0.01), and the least production in ATP was recorded at 825 nm at 5 J/cm^2^ (*p* < 0.0001).

ATP is present in all viable cells, and the luminescence signal is proportional to total ATP content, which may be influenced by other factors such as metabolic state, cell size, and differentiation status. Therefore, the observed increases in ATP levels indicate enhanced metabolic activity within the organoids, rather than a definitive increase in cell number. This distinction is particularly relevant in 3D neural organoid systems, where a heterogeneous population of proliferative progenitors and post-mitotic neurons coexists and contributes differently to overall metabolic output [11, 12, 28, 30]. Previous studies by Lee and co-workers (2023) and Malthiery and co-workers (2021) confirmed that green light between 520 and 532 nm significantly increases ATP levels during cellular proliferation studies [13, 31]. This coincides with the results obtained in this study showing increased cellular metabolic activity in NO-like structures during neural differentiation.

During neural differentiation, cells may exhibit increased metabolic demand without undergoing cell division, which can lead to elevated ATP levels independent of proliferation. Previous studies by Adan and co-workers have shown that ATP-based assays may overestimate proliferation when increases in signal reflect metabolic changes rather than actual cell division [28].

### Adaptive Response by △Ψm Mitochondrial Membrane Potential

**Fig. 4:**
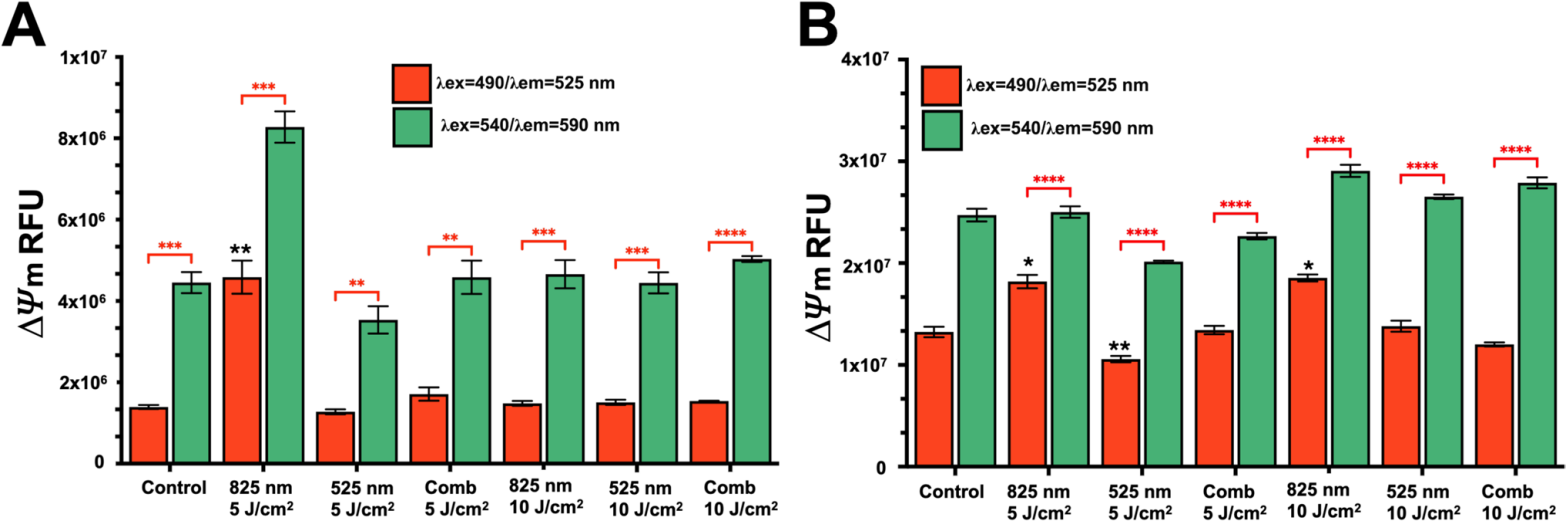
Assessment of mitochondrial membrane potential (MMP) using JC-10 dye on NO-like structures. **A** Quantitative analysis of JC-10 red and green fluorescence intensity at day 13, reflecting relative mitochondrial polarization. **B** Quantitative analysis of JC-10 red and green fluorescence at day 15, indicating changes in MMP over time. Data are presented as mean ± SEM from at least six independent experiments. Statistical significance was determined using two-way ANOVA with Tukey’s post-hoc test; *p* < 0.05 was considered significant. Red asterisks show comparison between red and green fluorescence, while black asterisks indicate comparison to the control. Statistical significance is indicated as *p < 0.5, **p < 0.01, ***p < 0.001, ****p < 0.0001

The analysis of the MMP (ΔΨm), a key indicator of mitochondrial health, is a crucial parameter in NO-like structures, reflecting the cell’s energy state and metabolic activity. By day 13 (Fig. 4A), data quantification showed a significant increase in MMP when NO-like structures were PBM treated with the 825 nm at 5 J/cm^2^ (*p* < 0.001) when compared to the control. By day 15 (Fig. 4B), significant increases in MMP relative to control were observed across most PBM conditions. The 825 nm treatment at 5 and 10 J/cm^2^ showed a strong increase (*p* < 0.05). In contrast, 525 nm at 5 J/cm^2^ continued to exhibit a significant decrease in MMP relative to the control (*p* < 0.01).

These findings suggest that PBM modulates mitochondrial function in a wavelength- and dose-dependent manner. An increase in ΔΨm is indicative of enhanced mitochondrial polarization and activity, which is associated with improved cellular bioenergetics and support for differentiation processes. PBM is known to stimulate mitochondrial respiratory chain activity, particularly cytochrome c oxidase, leading to increased electron transport and energy production [18, 32].

Interestingly, PBM-induced mitochondrial responses have been reported in mesenchymal stem cells derived from dental tissues, where enhanced mitochondrial activity was associated with neuronal differentiation and increased expression of neuronal markers [33]. These findings support the concept that mitochondrial activation acts as a key upstream regulator of PBM-mediated neural differentiation.

### Gene Expression Analysis on Differentiation

**Fig. 5:**
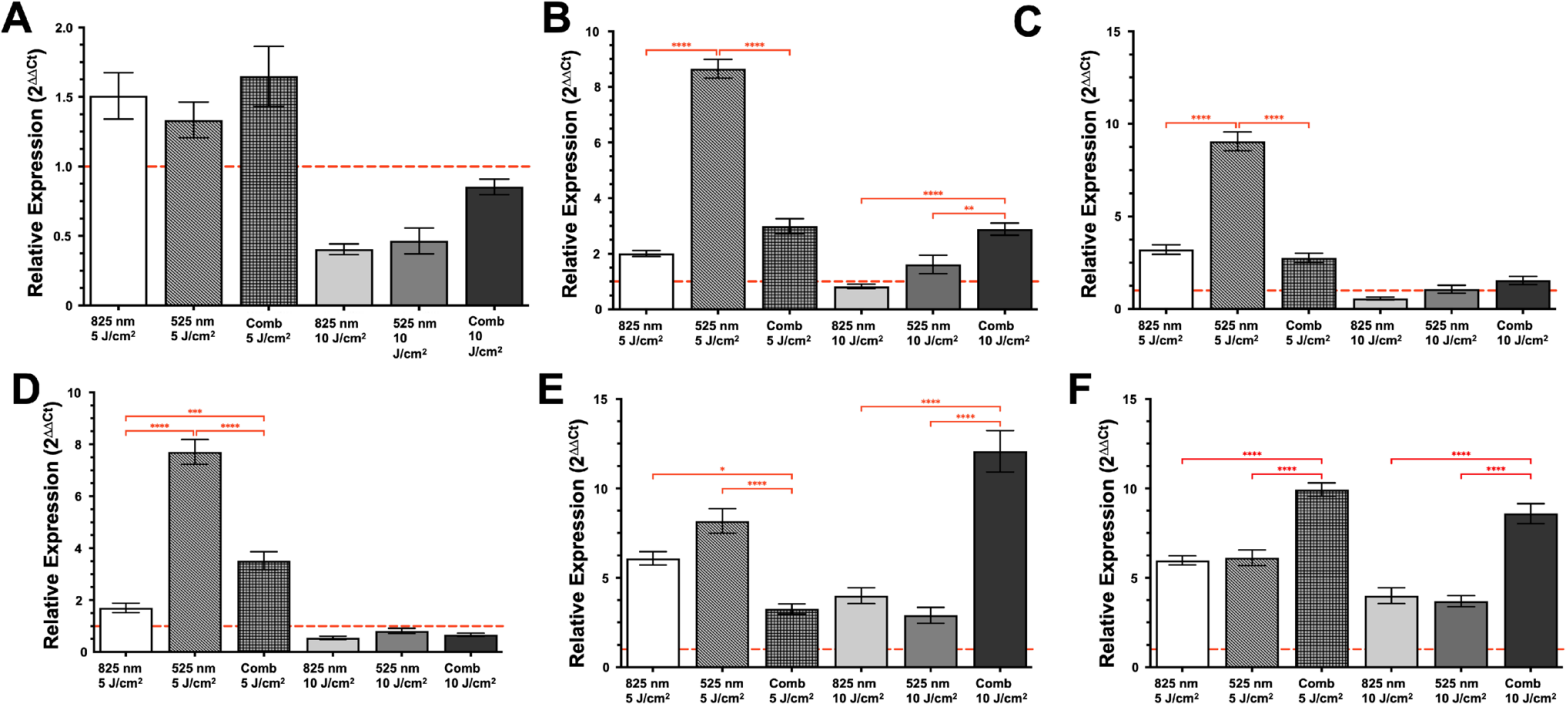
The relative expression of key genes associated with stemness and neural differentiation of **A** stem cell marker CD44, **B** *NES* (encoding Nestin) neuroepithelial marker, **C** *GFAP* (encoding glial fibrillary acidic protein) radial glial, **D** *TBR2* (encoding T-box brain protein 2) mature neuronal marker, **E** *TUBB3* (encoding β-tubulin III) neuronal marker beta, and **F** *RBFOX3* (encoding NeuN) mature neuronal marker at day 15 only. The red-dashed line indicates the baseline expression level of undifferentiated control ADMSCs, serving as a reference for relative gene expression changes following differentiation. Data are presented as mean ± SEM. Statistical significance is indicated as *p < 0.5, **p < 0.01, ***p < 0.001, ****p < 0.0001

To confirm successful neural lineage commitment prior to evaluating the effects of PBM, gene expression profiles of differentiated NO-like structures were interpreted relative to the baseline ADMSC state. Stem cell characterization was qualitatively assessed using stem cell surface markers CD 44 (Fig. 5A). Expression levels were elevated at 5 J/cm^2^, with the combination treatment showing the highest upregulation. Increasing the fluence to 10 J/cm^2^ significantly reduces CD44 expression across all wavelengths, approaching baseline.

Expression of neuroepithelial gene *NES* (encoding Nestin; Fig. 5B) showed a robust increase with 525 nm at 5 J/cm^2^, significantly exceeding both 825 nm and the combination treatment. At 10 J/cm^2^, *NSE* expression decreased substantially for 825 nm and 525 nm, while the combination maintains levels similar to those of 5 J/cm^2^.

Radial glial gene *GFAP* expression (Fig. 5C) was significantly increased in low fluence (5 J/cm^2^) treatment groups with 525 nm (~9-fold), showing the highest expression; the combination and 825 nm showed moderate induction. At high fluences (10 J/cm^2^), *GFAP* expression was reduced across all treatments groups.

Expression of the intermediate progenitor gene *TBR2* (Fig. 5D) was significantly upregulated in low-fluence groups of 5 J/cm^2^, 525 nm (~8-fold), combination (~4-fold), and 825 nm (~1.7-fold). In high-fluence groups of 10 J/cm^2^, *TBR2* expression levels across all groups remained low or downregulated.

The neuronal gene *TUBB3* (encoding β-Tubulin III; Fig. 5E) was increased and upregulated with the highest expression in combination 10 J/cm^2^ (~12-fold), followed by 525 nm 5 J/cm^2^ (~8-fold) treatment, consistent with early neuronal differentiation. Rolls and co-workers (2022) demonstrated that the neuronal marker β-Tubulin III exhibited delayed expression, being virtually undetectable at 18 days in vitro but emerging at 25 days in cells outside the neural rosettes. By day 53 and 81 in vitro, β-Tubulin III-positive neurons were clearly localized outside the rosette structures, indicating successful neuronal differentiation and migration of postmitotic neurons to basal regions [34]. However, the differential effects of PBM were observed to enhance the detection of β-Tubulin III by day 15 post-PBM treated groups. Similarly, the mature neuronal gene *RBFOX3* (encoding NeuN; Fig. 5F) showed notable upregulation across PBM-treated groups, particularly with the combination treatment, at 10 J/cm^2^, where it exceeds both single-wavelength conditions: 825 nm 10 J/cm^2^ (~4-fold), combination (825/525 nm) 5 J/cm^2^ (~10-fold), 525 nm 5 J/cm^2^ (~5-fold), and combination (825/525 nm) 10 J/cm^2^ (~9-fold). These increases reflect advanced neuronal differentiation and maturation, as mature neurons are typically post-mitotic.

The effects of PBM observed in this study may be associated with the activation of known intracellular signalling pathways involved in neurogenesis, including the Wnt/β-catenin, and MAPK/ERK pathways, which have previously been reported to regulate stem cell differentiation, survival, and neuronal maturation [20, 21, 35]. The observed increases in mitochondrial activity and neural marker expression are consistent with PBM-induced modulation of these signalling cascades, suggesting a potential mechanistic link between light stimulation and neural lineage commitment. Furthermore, PBM has been shown to enhance cellular metabolism and ATP production through mitochondrial stimulation, which may act upstream of these signalling pathways and contribute to the observed differentiation effects [17, 18, 24]. However, it is important to note that direct evaluation of these signalling pathways was not performed in the present study. Therefore, the proposed mechanisms remain speculative and are inferred from existing literature. Future studies should incorporate pathway-specific analyses, such as protein expression profiling, pathway inhibition assays, or phosphorylation studies, to confirm the involvement of these signalling networks in PBM-mediated neural differentiation within 3D culture systems. Therefore, while PBM appears to modulate cellular activity, additional proliferation-specific assays, such as Ki67 staining, EdU incorporation, or cell cycle analysis, would be required to confirm whether these effects are associated with changes in cell proliferation.

The generation of functional organoids is an important aspect in advancing the field of regenerative medicine. The morphology and size of brain organoids, the end product from NO, provide significant insight into the region of the brain generated. Kwak and co workers [36] stated that the initial NO measures approximately 350–400 µm, and this coincides with the results obtained. The differentiation of cells toward an NO lineage is verified by measuring the expression of key developmental markers. Early neural differentiation is initially characterized by the assessment of stem cell markers, which progressively decrease as cells commit to a neural fate, followed by an increase in neuroectodermal specification marker expression, such as SOX1 and SOX2. These early markers are primarily used to assess the self-organization and neural patterning of organoids, while maturation is subsequently confirmed by evaluating the expression of neuronal markers, including β-tubulin III, *RBFOX3* (NeuN), and MAP2. In addition, the expression of neurotransmitter-related and glial-associated markers, such as GFAP, has been reported in several studies and proposed as indicative of mature neuronal populations [36].

In the current study, qPCR analysis revealed changes in gene expression consistent with neural differentiation and maturation. A reduction in the stem cell marker CD44, together with a significant upregulation of neuroepithelial and progenitor markers, such as Nestin and radial glial marker GFAP, indicates early neural commitment and organoid self-organization. Furthermore, increased expression of neuronal lineage markers, TBR2 and β-Tubulin III, supports progression toward neuronal differentiation. The enhanced expression of the mature neuronal marker NeuN, particularly under combination treatment, suggests advancement toward terminal neuronal maturation.

Despite the promising findings, several limitations should be acknowledged. ATP measurements reflect cellular metabolic activity rather than direct proliferation and therefore cannot distinguish between changes in cell number, size, or metabolic state. In addition, cell viability was assessed qualitatively, and future studies should incorporate quantitative assays to strengthen interpretation. The structures generated in this study are described as neuronal organoid-like structures, as functional validation and full cellular complexity of mature organoids were not assessed. Furthermore, gene expression analysis was limited to a selected panel of markers which may not fully capture the dynamic progression of neural differentiation. Finally, the molecular mechanisms underlying PBM effects, including the involvement of Wnt/β-catenin, PI3K/Akt, and MAPK/ERK pathways, were not directly investigated and remain to be confirmed in future studies.

## Conclusion

This study demonstrates a protocol for generating neuronal organoid-like structures and explores the potential of PBM as a modulatory tool for translational applications. PBM may exert dose-dependent influence on neural differentiation; at low-fluence irradiation (5 J/cm^2^), particularly at 525 nm, it supports stem cell maintenance and early-to-intermediate neural differentiation, as evidenced by the upregulation of progenitor and early neuronal markers. In contrast, higher fluence (10 J/cm^2^) generally attenuates early differentiation; however, when applied as a combined wavelength treatment, it appears to promote late-stage neuronal maturation, marked by increased NeuN expression. Notably, the decrease in ATP levels under combined high-fluence conditions, alongside increased organoid size and preserved mitochondrial membrane potential (ΔΨm), suggests a shift from metabolically active states toward cellular maturation and structural organization, rather than reduced viability or function. These findings highlight the importance of interpreting PBM effects through an integrated framework that considers metabolic, mitochondrial, structural, and gene expression changes collectively. Furthermore, the differential responses observed support the concept of tailoring PBM parameters to regulate specific stages of tissue regeneration. PBM represents a promising non-invasive strategy for directing stem cell fate within 3D systems, although further functional and mechanistic studies are required to fully elucidate its effects.

## Data Availability

The raw and analysed data used to support the findings of this study are available from the corresponding author upon request.
